# Effect of DNA Methyltransferase in Comparison to and in Combination with Histone Deacetylase Inhibitors on Hepatocellular Carcinoma HepG2 Cell Line

**DOI:** 10.31557/APJCP.2019.20.4.1119

**Published:** 2019

**Authors:** Masumeh Sanaei, Fraidoon Kavoosi

**Affiliations:** Research Center for Non-Communicable Diseases, Jahrom University of Medical Sciences, Jahrom, Iran. *For Correspondence: Kavoosifraidoon@gmail.com

**Keywords:** Genistein, valproic acid, apoptosis, proliferation, hepatocellular carcinoma

## Abstract

**Background:**

DNA demethylating agents and histone deacetylase inhibitors can affect reactivation of gene expression and apoptosis induction by DNA acetylation and demethylation. The aim of the present study was to analyze the effects of DNA demethylating agent genistein (GE) and histone deacetylase inhibitor valproic acid VPA), alone and combined, on hepatocellular carcinoma Hep G2 cell line.

**Methods:**

The cells were treated with various doses of genistein and valproic acid (alone and combined) and the MTT assay and flow cytometry were used to determine cell viability and apoptosis.

**Results:**

Genistein and valproic acid inhibited the growth of HepG 2 cells significantly. Result of flow cytometry demonstrated that genistein and valproic acid (alone and combined) induce apoptosis significantly in a timedependent manner.

**Conclusions:**

Genistein and valproic acid can significantly inhibit proliferation and induce apoptosis in HepG2 cell line. The apoptotic effects of GE in combination with VPA were more significant that of each compound alone.

## Introduction

Eukaryotic chromatin is the state in which DNA is packaged within the cell. The nucleosome is the basic packing unit of the eukaryotic genome contains an octamer of the four core histones (H3, H4, H2A, H2B) around which 147 base pairs of DNA are wrapped (Kouzarides., 2007).

In the normal cells, the pattern of DNA methylation is conserved after DNA replication and cell division through the methylation of cytosine by a maintenance DNA methyltransfrase 1 (DNMT1). DNA methylation of genes occurs in the promoter region that contains CpG islands. The methylation of this region silences gene expression and is a normal event that occurs in the cells to regulate gene expression but aberrant DNA methylation of tumor suppressor genes occurs in cancers. On the other hand, the aberrant methylation of tumor suppressor genes leading to the cancers (Costello et al., 2000). 

Histone acetylation, which is done by histone acetylases, is one of the other posttranslational modifications of the histones occurs in the amino groups of all core histones (H3 and H4 H2A and H2B). In fact, acetylation is a key component in the regulation of gene expression (Wade., 2001). 

It should be noted that histone deacetylases are well characterized cellular oncogenes and an aberrant recruitment of these enzymes leading to tumorigenesis (Barneda-Zahonero et al., 2012; Song et al., 2000). Genistein (GE), belongs to flavonoids, has an inhibitory effect on tumorigenesis through epigenetic regulations. This compound has anticancer property and activates tumor suppressor genes by modulating chromatin configuration and DNA methylation (Banerjee et al., 2008; Kikuno et al., 2008; Sarkar et al., 2003). Several studies have reported that GE has effect on different cancer cell lines by affecting various cellular targets, including tumor suppressor genes, cancer cell growth inhibition (Luo et al., 2008; Yu et al., 2005; Zhang et al., 2011), apoptosis induction (Wang et al., 2011; Ullah et al., 2011) and cell cycle arrest (Bielecki et al., 2011; Han et al., 2010; Rahal et al., 2010). It has been reported that low physiological concentrations of genistein have an anticancer effect in HCT116 colon cancer cells (Mure et al., 2006) and can alter DNA methylation consistent with altered gene expression in breast cancer cell lines (King-Batoon et al., 2008).

Another study has reported that GE can inhibit tumorigenesis through epigenetic control in several cancer cell lines (Li et al., 2013; Li et al., 2009) and induce a chemopreventive effect against various types of cancer cells, including cancer of prostate, colon and esophageal cancer (Barnes et al., 1995) by demethylating activity leading to reactivation of methylation-silenced genes (Fang et al., 2007).

In vitro study has demonstrated that GE inhibits the proliferation of ovarian SKOV3 cells by genetic regulation of the cell cycle or apoptosis in a dose and time dependent manner (Shan et al., 2012) and also induces chromatin decondensation and histone acetylation in MCF-7 breast cancer cell line (Marchion et al., 2005).

Our previous studies clearly show that genistein has a significant inhibitory effect on the growth of hepatocellular carcinoma HepG2 cell lines and induces apoptosis in these cell line with a time-dependent manner (Sanaei et al., 2017a; Sanaei et al., 2017b). 

Alterations in HDAC activity have been observed in numerous cancers (Cress et al., 2000; Urnov et al., 2008; Mahlknecht et al., 2000; Timmermann et al 2001). Several studies have reported that histone deacetylase inhibitors, as anticancer agents, are used for the treatment of solid and hematological cancers (Vigushin et al., 2003; Melnick et al., 2002; Marks et al., 2001). These compounds induce reactivation of tumor suppressor genes that have been silenced during the course of neoplastic transformation (Roth et al., 2001). 

Histone deacetylase inhibitor valproic acid (VPA) suppresses transcription factors which recruit histone deacetylases. It causes hyperacetylation of the N- terminal tails of H3 and H4 in vivo and in vitro (Göttlicher et al., 2001). Our previous work indicated that VPA significantly inhibit the growth and induce apoptosis in the HT 29 cell line (Sanaei et al., 2016). However, to our knowledge, there have been no reports on the effects of GE alone or in combination with VPA on hepatocellular carcinoma HepG 2 cell line. 

Therefore, we decided to investigate the effects of these compounds (alone and combined) on proliferation and apoptosis of hepatocellular carcinoma HepG 2 cell line.

## Materials and Methods

Human hepatocellular carcinoma cells (HepG2) were purchased from the National Cell Bank of Iran-Pasteur Institute and maintained in Dulbecco minimal essential medium (DMEM) containing 100 mL/L fetal bovine serum (FBS), 100U/mL penicillin, 100 U/mL streptomycin at 37^o^C in a humidified atmosphere containing 50 mL/L CO_2_. VPA was obtained from Sigma and dissolved in phosphate-buffered saline (PBS), PBS was added to culture medium as a negative control. GE, DMEM and MTT (3-[4, 5-dimethyl-2-thiazolyl]-2, 5-diphenyl-2Htetrazolium bromide) were purchased from Sigma (Sigma, St. Louis, MO). GE dissolved in dimethyl sulfoxides (DMSO), DMSO was added to culture medium as a negative control (Kavoosi et al., 2016). All other chemicals were obtained from the best sources available.

*Cell Culture and Determination of IC50 Value by MTT Assay*

 The cells were cultured and grown in in DMEM containing 100μl /L FBS. The cultures were incubated at 37˚c in a humidified incubator containing 5% CO_2_, 95% ambient air. When cells became >80% confluent, 5 × 10^5^ cells (HepG2) were seeded into 96-well plates (Becton-Dickinson) for 24 h in DMEM culture medium before incubation with certain concentrations of GE (0, 10, 25, 50 and 100 μM/lit), which was dissolved in DMSO; DMSO was present at 0.01–0.3% in the medium. After 24 h, culture medium was changed with culture medium contains various concentrations of GE. On days 2 and 3 after treatment with GE (24 and 48h after treatment), MTT assay was done. The MTT assay for determination of IC_50_ values for VPA with certain concentrations of VPA (0, 1, 5 and 10 μM/lit) which was dissolved in phosphate-buffered saline (PBS) was done. 

We observed the cells morphous under light microscope every day. After the termination of the culture, MTT (0.5 mg/mL) was added for an additional 4 hours. Absorbance at a wavelength of 570 nm was determined for each well using a microplate ELISA reader. So we can get the optical density (OD) of every well. Inhibition ratio of different concentrations was calculated.

*Determination of Cell Viability by MTT Assay*

The MTT assay was commonly used to assess cell proliferation and viability by measuring the reduction of yellow MTT by mitochondrial dehydrogenases in viable cells (Spinner, 2000). This yields purple formazan crystals that detected colorimetrically at 570 nm. To determine the effect of GE and VPA, the cells were seeded in triplicate in 24-well plates and treated with GE (25 µM, IC_50_) and VPA (5 µM, IC50) in different period times (24 and 48 h), we selected VPA doses according to IC50 and our previous work 65, 67). After 4 h of exposure at 37°C, the formazan crystals were dissolved in dimethylsulfoxide (DMSO). Using an ELISA reader (TECAN Safire II, Sweden) the absorbance was measured at 570 nm.

*Determination of Apoptotic Cells by Flow Cytometry Assay*

To determine the apoptotic cells, the flow cytometry assay was done (Darzynkiewicz et al., 2001).

The cells were cultured in 24-well culture plates and divided into different experimental groups after 24 h. Two groups received a single dose of GE at the concentration of 25 μM, two groups received a single dose of VPA at the concentration of 5μM, two groups received GE (25μM, IC_50_) combined with VPA (5μM, IC50) for 24 and 48 h respectively. Control groups received DMSO and FBS as control groups. After the termination of the culture, all the adherent cells were collected with 0.05% trypsin, washed with cold phosphate-buffered saline (PBS) and re suspended in Binding buffer (1x). After addition of AnnexinV-FITC and propidium iodide (PI, Becton-Dickinson, San Diego, CA), analysis was carried out according to the manufacturer’s protocol (BMS500F1/100CE AnnexinV-FITC, eBiscience, USA). Finally, the apoptotic cells were counted by FACS can flow cytometry (Becton Dickinson, Heidelberg, Germany). All experiments were processed independently three times. A minimum of 5×10^5^ cells/ml were analyzed for each sample.

*Statistical Analysis*

The database was set up with the SPSS 16.0 software package for analysis. The data were obtained from three independent experiments and are presented as means ± SD (standard deviations). Statistical comparisons between groups were performed with ANOVA (one-way ANOVA) and Tukey test. A significant difference was considered as P 0.05.

## Results

*Result of Determination of IC50 Value by MTT Assay*

**Figure 1:**
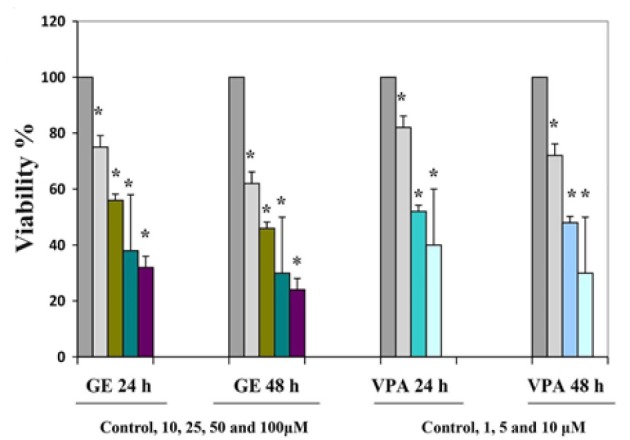
Effect of GE and VPA on the Viability of Hepatocellular Carcinoma Cell Line Determined by MTT Assay. The cells were treated without and with different concentrations of GE and VPA for 24 and 48 h, the control groups received DMSO only. Each experiment was conducted in triplicate. Mean values from the three experiments ± standard error of mean are shown. Asterisks (*) indicate significant differences between treated cell groups and the control groups (*P < 0.001)

The effects of the GE and VPA on the cell viability after exposure with various concentrations (as mentioned) were assessed by MTT assay. Dose- and time-dependent antiproliferative effects were observed with IC50_s_ for GE and VPA (Figure 1). Reduction of cell viability by 50% (IC50) required 25 μM GE for GE-treatment groups and 5 μM VPA for VPA-treatment groups at different times (24 and 48h).

*Result of Determination of Cell Viability by MTT Assay*

**Figure 2:**
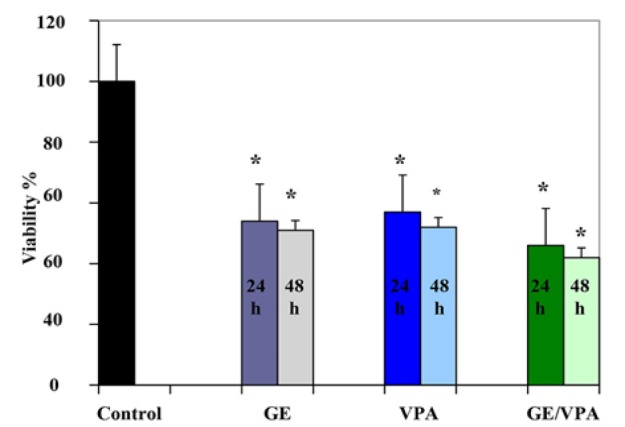
Effect of GE and VPA (Alone and Combined) on Human Hepatocellular Carcinoma HepG2 Cell Proliferation. HepG2 cells were treated with GE (25 μM/L), VPA (5 μM/L) and GE/VPA (25/5 μM) for 24 and 48h, the control groups received DMSO only. Cell survival was determined by the MTT assay. Data are presented as mean ± standard error of the mean from at least three different experiments. Asterisks (*) indicate significant differences between treated cells and the control group. *P < 0.01 as compared to the control group

The cell vitality in the cells, which treated with GE (25µM, IC50) and VPA (5 µM, IC50) alone and also GE combined with VPA in different time periods (24 and 48h) were analyzed by using the MTT assay. The amounts of reduced MTT in the all groups treated with GE and VPA were significantly lower than that of the control group (P 0.001). The Percentage of the cell viability for GE (25μM)-treatment groups were 54 and 51 % (P < 0.001) and for VPA (5μM)- treatment groups were 57 and 52 % (P < 0.001) at different time periods (24 and 48h) respectively. The percentage of the cell viability for GE combined with VPA treatment groups were 46 and 42 % at different time periods (24 and 48h) respectively. There was a significant difference between the percentage of cell viability of all experiment groups with control groups (Figure 2). There was a significant difference between the percentages of the cell viability of single agent treatment and combination treatment groups, between GE (24h) and GE/VPA (24 and 48 h) groups (P < 0.03), between GE 48h and GE/VPA 48 h groups (P < 0.013), between VPA 24h and GE/VPA 24 and 48 h groups (P < 0.002) and between VPA 48h and GE/VPA 48 h groups (P < 0.006).

*Result of Determination of Apoptosis by Flow Cytometry Assay*

**Figure 3:**
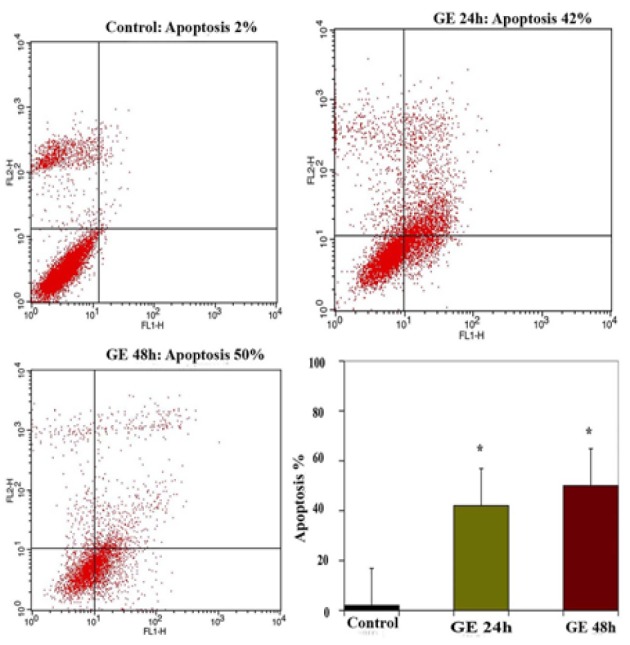
The apoptosis-inducing effect of GE was investigated by flow cytometric analysis of HepG2 cells. The cells were treated with GE (25 μM) for 24 and 48 h, except the control groups received DMSO only, and the apoptosis inducing effect of GE was investigated by flow cytometric analysis, the cells stained with Annexin V and propidium iodide. The results were obtained from three independent experiments and were expressed as mean ± standard error of the mean. Asterisks (*) indicate significant differences between treated cells and the control group. *P < 0.001, n = 3

**Figure 4:**
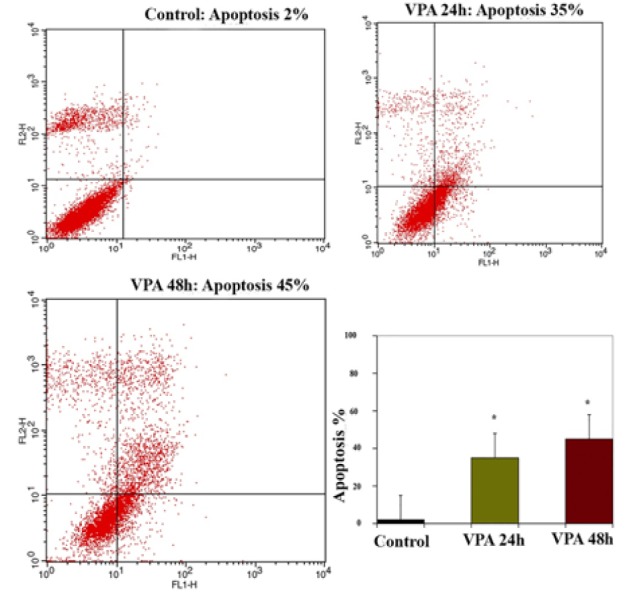
The Apoptosis-Inducing Effect of VPA (5μM) was Investigated by Flow Cytometric Analysis of HepG2 Cells after Treatment Times (24 and 48 h) Stained with Annexin V and Propidium Iodide, the Control Groups Received DMSO Only. The results were obtained from three independent experiments and were expressed as mean ± standard error of the mean. Asterisks (*) indicate significant differences between treated cell groups and the control group. *P < 0.001, n = 3

**Figure 5:**
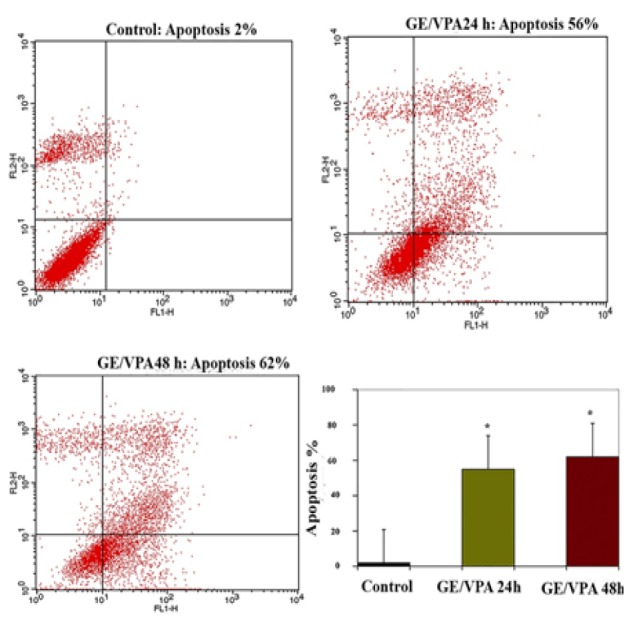
The Apoptosis-Inducing Effects of GE (25 μM) and VPA (5 μM) were Investigated by Flow Cytometric Analysis of HepG2 Cells (as Described in the Methods) Stained with Annexin V and Propidium Iodide, the Control Groups Received DMSO Only. The combination of GE and VPA induced cell apoptosis in HepG 2 cells significantly. Asterisks (*) indicate significant differences between treated cells and the control group. Results were obtained from three independent experiments and were expressed as mean ± standard error of mean n = 3

**Figure 6:**
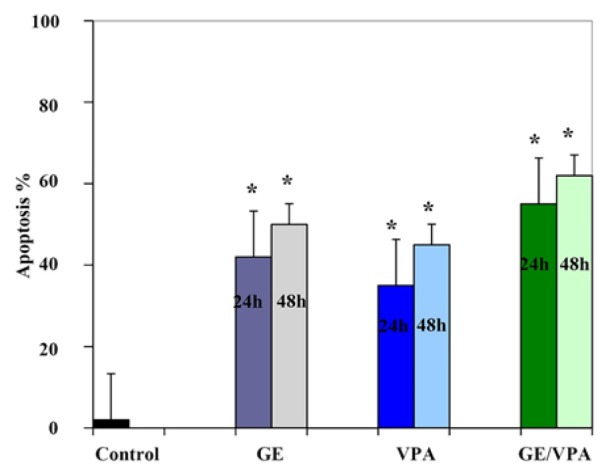
Comparative Analysis Shows that the Apoptotic Effects of GE Combined with VPA are more Significant than of Each Compound Alone

The apoptosis-inducing effect of GE and VPA alone and combined were investigated by flow cytometric analysis of HepG2 cells stained with Annexin V and propidium iodide. We observed via flow cytometry that these compounds induce apoptosis in this cell line significantly. The percentage of apoptotic cells in GE (25μM, IC50)-treatment groups at different time periods (24 and 48h) were 42 and 50 % (Figure 3), and in the VPA (5μM)-treatment groups at different time periods (24 and 48h) were 35 and 45 % respectively (Figure 4). The percentage of apoptotic cells in the combined treatment groups were 55 and 62 % in different time periods (24 and 48h) respectively (Figure 5). The apoptotic effects of GE in combination with VPA were more significant that of each compound alone (Figure 6).

## Discussion

Acetylation of the histones is a posttranslational modification found in the all animal species. This process occurs at specific lysine residues, all of which occur in the amino-terminal domains of the core histones. Acetylation and deacetylation of histone proteins play a major role in the regulation of gene expression (Grunstein et al., 1997). There are two classes of enzymes involved in the acetylation of histones including histone acetyl transferases (HATs) and histone deacetylases (HDACs). 

Several studies have shown that altered HAT or HDAC activities are associated with different cancers (Muraoka et al., 1996; HeLZ., 1998; Grignani et al., 1998; Lin et al., 1998). Addition and removal of acetate groups (acetylation and deacetylation) are catalyzed by these enzymes (Turner, 1991). Histone deacetylases (class I and class II) involve in deacetylation of the histone and can be inhibited by a diverse group of compound such as histone deacetylase inhibitors (Marks et al., 2003). Histone deacetylase inhibitors (HDACi) are anti-cancer drugs which induce chromatin remodelling and alter gene transcription by which induces tumor cell apoptosis, cell cycle arrest, cell differentiation, modulation of immune responses and altered angiogenesis. Besides, these compounds can augment the apoptotic effects of other anti-cancer agents by diverse molecular targets (Frew et al., 2009). VPA relieves HDAC-dependent transcriptional repression and causes hyperacetylation of histones in vivo and in vitro. This compound inhibits tumor growth and metastasis formation in animal experiments (Machado et al., 2011). 

We report that VPA inhibits cell growth and induces apoptosis in hepatocellular carcinoma HepG2 cell line significantly. Similar to our report, it has been shown that VPA suppresses tumor cell proliferation in a dose-dependent manner in human hepatocellular cancer cells (HUH7) in vitro (Machado et al., 2011). It has also been shown that VPA induces apoptosis, upregulates P21/Waf1/CIP1, represses TMPRSS2-ERG expression and affects the acetylation status of p53 in ERG-positive prostate cancer cells (Fortson et al., 2011). On the other hand, VPA inhibits proliferation of ovarian cancer SKOV3 cells in a dose and time dependent fashion (Shan Z et al., 2012).

Various molecular pathways have been reported for HDACI. It has shown that histone deacetylase inhibitors upregulate the intrinsic apoptosis pathway via proapoptotic genes Bmf38 and Bim induction (Zhao et al., 2005). These compounds disrupt the function of HSP90 protein chaperone by inducing hyperacetylation of this protein that normally protects cancer-related proteins from degradation (Kovacs et al., 2005). In vitro study has demonstrated that VPA induces histone (H3 and H4) hyperacetylation, activates p21, restore p16/CDK4 pathway and suppresses CMYC oncogene in medulloblastoma (DAOY and D283-MED) cell lines (Li et al., 2005). Other studies have indicated that VPA modulates expression of p21WAF1/CDKN1A by which induces cell cycle arrest in G1/S phase (Cheng et al., 2007) and also induces Fasdependent apoptosis associated with an overexpression of Fas and Fas ligand (Angelucci et al., 2006). 

Our work indicated that GE with concentration of 25 μM induces significant apoptosis in HepG2 cell. We have previously shown that GE (25μM, IC50) inhibits the growth of hepatocellular carcinoma PLC/PRF5 cells significantly with a time and dosedependent manner (Dastjerdi et al., 20154). 

A similar study has demonstrated that GE inhibits the viability of human colon cancer HT-29 cell via induction of apoptosis mainly through regulation of p21WAF1 and Bax/Bcl-2 expression (Yu et al., 2004). Furthermore, GE inhibits the growth of MDA-MB-231 breast cancer cells, regulates the expression of apoptosis-related genes, and induces apoptosis through a p53-independent pathway (Li et al., 1999) and also dietary genistein inhibits prostate cancer metastasis (Lakshman et al., 2008). Our investigation on the apoptotic effect of GE is not supported by a few studies. It has been reported that GE can act as an estrogen agonist in vivo and in vitro, resulting in the proliferation of cultured human breast cancer MCF-7 cells (Hsieh et al., 1998). 

In another report, biphasic effect of GE on prostatic cancer cell has been observed (El Touny et al., 2009). The results of the present study showed that apoptotic and antiprolifrative effects of GE combined with VPA were more significant than of each compound alone. Consistent with our findings, it has been reported that treatment with methyltransferase inhibitor 5-azacitidine (aza-CR) combined with histone deacetylase inhibitor sodium phenylbutyrate induce apoptosis in myeloid leukemia cell significantly (Gore et al., 2006). Furthermore, it has been demonstrated that 5-aza-2´-deoxycytidine combined with histone deacetylase inhibitor sodium phenylbutyrate induce apoptosis in lung tumor cell significantly (Belinsky et al., 2003). 

Different mechanisms and pathways have been reported for GE, it induces cell cycle arrest in the G0/G1 and G2/M phases in Bel 7402 hepatocellular carcinoma cells (Gu et al., 2005). In vitro study has indicated that GE activates several endoplasmic reticulum stress-relevant regulators such as GADD153, m-calpain, caspase-12 and GRP78 in hepatocellular carcinoma Hep3B cells (Yeh et al., 2007). It has been shown that GE can reactivate methylation-silenced tumor suppressor BTG3 gene by CpG demethylation and inhibition of DNMT and MBD2 activity in HEK- 293 renal cell carcinoma (Majid et al., 2009). 

GE can reverse DNA hypermethylation and reactivates p16INK4a, RARb and MGMT in KYSE 510 esophageal squamous cell carcinoma cells, KYSE 150 cells, prostate cancer LNCaP and PC3 cells (Fang et al., 2005). GE can also induce methylated BTG3 promoter expression in human prostate carcinoma cell lines (LNCaP, PC3), increase levels of acetylated histones 3, 4, histone 3 trimethylated at lysine 4, histone 3 dimethylated at lysine 4, RNA polymerase II and decrease DNA methyl transferase and methyl-binding domain protein 2 activity, and increase histone acetyl transferase (HAT) activity (Majid et al., 2010). 

In this experiment, We did not perform western blot in order to evaluate of HDAC1 and HDAC2 expression and also the acetylation status of histone H3 and a-tubulin but we decide to perform them in the next work. Previously, we evaluated the effect of GE and VPA on the other HCC cell lines including PLC/PRF5 and HepG 2 (Sanaei et al., 2017a; Kavoosi et al., 2016; Sanaei et al., 2017b). Thus, our studies provide a new avenue for cancer treatment through demethylating agents combined with histone deacethylase inhibitors.

In conclusion, GE and VPA (alone and combined) can inhibit the growth of HCC in vitro which may provide a novel approach for the prevention and open a new window to find the new strategy for cancer prevention.

## Conflicts of interest

The authors report no conflicts of interest in this work.
